# Are central and systemic inflammation associated with fatigue in cerebral small vessel disease?

**DOI:** 10.1177/17474930241245613

**Published:** 2024-04-12

**Authors:** Amy A Jolly, Robin B Brown, Daniel J Tozer, Young T Hong, Tim D Fryer, Franklin I Aigbirhio, John T O’Brien, Hugh S Markus

**Affiliations:** 1 Stroke Research Group, Department of Clinical Neurosciences, University of Cambridge, Cambridge, UK; 2 Department of Clinical Neurosciences, University of Cambridge, Cambridge, UK; 3 Wolfson Brain Imaging Centre, Department of Clinical Neurosciences, University of Cambridge, Cambridge, UK; 4 Department of Psychiatry, University of Cambridge, Cambridge, UK

**Keywords:** Cerebral small vessel disease, fatigue, inflammation, positron emission tomography imaging, microglia, stroke

## Abstract

**Background:**

Fatigue is a common symptom in cerebral small vessel disease (SVD), but its pathogenesis is poorly understood. It has been suggested that inflammation may play a role. We determined whether central (neuro) inflammation and peripheral inflammation were associated with fatigue in SVD.

**Methods:**

Notably, 36 patients with moderate-to-severe SVD underwent neuropsychometric testing, combined positron emission tomography and magnetic resonance imaging (PET–MRI) scan, and blood draw for the analysis of inflammatory blood biomarkers. Microglial signal was taken as a proxy for neuroinflammation, assessed with radioligand ^11^C-PK11195. Of these, 30 subjects had full PET datasets for analysis. We assessed global ^11^C-PK11195 binding and hotspots of ^11^C-PK11195 binding in the normal-appearing white matter, lesioned tissue, and combined total white matter. Peripheral inflammation was assessed with serum C-reactive protein (CRP) and using the Olink cardiovascular III proteomic panel comprising 92 biomarkers of cardiovascular inflammation and endothelial activation. Fatigue was assessed using the fatigue severity scale (FSS), the visual analog fatigue scale, and a subscale of the Geriatric Depression Scale.

**Results:**

Mean (SD) age was 68.7 (11.2) years, and 63.9% were male. Of these, 55.6% showed fatigue on the FSS. Fatigued participants had higher disability scores (*p* = 0.02), higher total GDS scores (*p* = 0.02), and more commonly reported a history of depression (*p* = 0.04). ^11^C-PK11195 ligand binding in the white matter was not associated with any measure of fatigue. Serum CRP was significantly associated with average fatigue score on FSS (ρ = 0.48, *p* *=* 0.004); this association persisted when controlling for age, sex, disability score, and depression (β = 0.49, 95% CI (0.17, 2.26), *p* = 0.03). Blood biomarkers from the Olink panel showed no association with fatigue.

**Conclusion:**

In symptomatic SVD patients, neuroinflammation, assessed with microglial marker ^11^C-PK11195, was not associated with fatigue. We found some evidence for a role of systematic inflammation, evidenced by an association between fatigue severity and raised CRP, but further studies are required to understand this relationship and inform whether it could be therapeutically modified to reduce fatigue severity.

**Data access statement:**

Data for this study are available from the corresponding author upon reasonable request.

## Introduction

Fatigue is a common symptom following stroke and has been identified as a major research priority by patient panels.^1^ A recent global review suggested that it affected half of all stroke patients.^2^ Despite its importance, the mechanisms underlying fatigue are unclear, and there are few proven effective therapies.^3^

Multiple mechanisms have been suggested to contribute to post-stroke fatigue (PSF).^4^ These include predisposing factors, such as pre-stroke fatigue or depression, neurobiological factors, including lesion location and neuroendocrine changes, and perpetuating factors, including affective disorders and reduced physical activity.^4,5^ One additional factor that has been increasingly implicated is inflammation, both systemic and central.^4,5^ Stroke induces a systemic inflammatory response, and it has been suggested that this may increase fatigue, although few studies have examined the association.^5–7^ In one of these, baseline interleukin (IL)-1β levels were associated with PSF 6 months after stroke, whereas baseline IL-1ra and IL-9 levels negatively correlated with PSF at 12 months.^8^ Increased C-reactive protein (CRP) levels have been associated with PSF in some^7^ but not all studies.^9^

Systemic inflammatory factors may mediate increases in fatigue through an exaggerated central nervous system (CNS) inflammatory response,^10^ although there is little data on whether fatigue is associated with increased CNS inflammation.

One stroke condition in which fatigue seems particularly common is cerebral small vessel disease (SVD).^11^ This causes acute lacunar stroke and more diffuse chronic white matter changes. Fatigue is a common symptom in both acute and chronic SVD.^11^ To better understand the role of inflammation in PSF, we determined whether CNS and peripheral inflammation were associated with fatigue in symptomatic SVD patients.

## Methods

### Study design

This study was embedded in the MINocyclinE to Reduce inflammation and blood–brain barrier leakage in small Vessel diseAse (MINERVA) trial (ISRCTN: 15483452)—a phase II, double-blind and placebo-controlled trial—testing whether minocycline (100 mg) can reduce neuroinflammation in SVD.^12^ In the current study, only baseline data, prior to administration of any study treatment, were analyzed.

### Participants

Inclusion criteria for the MINERVA study were those aged above 18 years with evidence of SVD as manifested by lacunar stroke, cognitive impairment, or gait apraxia in addition to confluent white matter hyperintensities (WMH) on magnetic resonance imaging (MRI) (Fazekas score of ⩾2).^12,13^ Participants were recruited at least 3 months after stroke to prevent acute effects of stroke impacting inflammatory measures.^12^ Exclusion criteria included dementia, lack of capacity to consent, strokes of any other cause than SVD, cortical strokes, lacunar infarcts ⩾1.5 cm, probable cerebral amyloid angiopathy (CAA) based on modified Boston criteria,^14^ suspected/known monogenic SVD, and contraindication to MRI, positron emission tomography (PET), or minocycline.^12^

During the period 29 March 2019 to 15 June 2022, 44 participants were randomized into the MINERVA study. Presenting symptoms were lacunar stroke in 41 and cognitive impairment in 3 participants. The fatigue severity scale (FSS) was added after 8 patients had been recruited; therefore, 36 completed fatigue testing at baseline and were included in the current analyses.

### Ethical approvals

The MINERVA study was approved by the East of England—Cambridge Central Ethics Committee (18/EE/0237) on 15 November 2018. All participants gave written informed consent.

### Measures

#### Demographics

Demographics of the cohort were collected, including age, sex, modified Rankin Scale (mRS), disability score, and cardiovascular risk factors (CVRFs). Medical history, including history of previous strokes or transient ischemic attack (TIA), and history of depression were also recorded.

#### Neuropsychometry

A neuropsychological battery was carried out at baseline, including cognitive and mood testing.^12^ Relevant neuropsychiatric measures are discussed below.

The FSS is a nine-item self-report scale assessing the impact of fatigue on activities of daily life over the past week.^15^ Participants rate how much they agree with nine statements on a scale from 1 to 7 (*strongly disagree* to *strongly agree*).^15^ An average score of ⩾ 4 across all questions has been used to indicate significant fatigue.^16,17^ The FSS also included the visual analog fatigue (VAF) scale, a single question asking participants to rate their global daily fatigue on a scale of 0 (*worst symptom*) to 10 (*normal*).

The Geriatric Depression Scale (GDS) is a self-report measure of depression validated and used in SVD cohorts.^18–20^ Higher scores indicate higher depression; a score of 0–10 is within the normal range, while 10–19 and 20–30 show mild and severe depressive symptoms, respectively.^20^ The GDS can be broken down into three subscales: apathy, anxiety, and fatigue.^21^ The fatigue subscale (GDS-F) has a maximum score of 9 and has been validated against existing fatigue measures; higher scores denote higher fatigue.^21^

#### PET–MRI

Full methods for PET–MRI acquisition and processing have been described previously.^12,22^ Briefly, participants completed combined PET–MRI at baseline using a 3T GE SIGNA PET–MRI scanner at the Wolfson Brain Imaging Center (WBIC; Cambridge, UK).^12^ PET data were collected for 75 min after participants were injected with a dose of ^11^C-PK11195 (PK), produced at the WBIC’s Radiopharmaceutical unit.^12,22^ PK binds to the 18 kDa translocator protein (TSPO), with higher TSPO levels thought to indicate higher microglial activation.^23–25^ We assessed the binding potential (BP) of PK to TSPO within the white matter tissues of the brain using a single reference tissue model corrected for intravascular binding.^26^ Measures of PK BP were determined relative to the white matter of a library of healthy control participants for total white matter (AWM), normal-appearing white matter (NAWM), and lesioned white matter tissue. Masks that were eroded by 3 mm to prevent any possible contamination from gray matter or ventricles^22^ were also used, resulting in additional PK binding measures for eroded AWM (EAWM) and eroded NAWM (ENAWM). In total, 30 subjects had full PET datasets for analysis.

#### MRI

Simultaneous to PET-imaging, non-contrast MRI was carried out, including 3D T1-weighted, T2*-weighted gradient-echo and arterial spin labeling, fluid-attenuated inverted recovery (FLAIR), and diffusion tensor imaging (DTI) sequences.^12^ Brain atrophy was assessed using the SIENAX pipeline from the FMRIB Software Library (FSL), in which brain volume is extracted from T1 sequences and segmented into volumes of white matter, gray matter, and total brain volume (https://fsl.fmrib.ox.ac.uk/fsl/fslwiki/).^27,28^ Skull size was accounted for with a scaling factor.^27,28^ WMHs were identified as hyperintense areas on FLAIR imaging and rated using the semi-automated contouring tool within the Jim analysis software (version 8, Xinapse Systems, http://www.xinapse.com/j-im-8-software), and WMH volumes were multiplied by the scaling factor produced by SIENAX to account for skull size.

Cerebral microbleeds (CMBs) were identified as small round hypodense dots as seen on T2*-weighted GE sequences. Lacunes were defined as round hypodense areas between 3 and 15 mm on T1-weighted and FLAIR sequences.^29^ A trained rater assessed WMH volume, CMB, and lacune count (R.B.B.).

#### Blood biomarkers

Blood biomarkers were collected at baseline. Serum CRP was measured using enzyme-linked immunosorbent assay (Siemens Healthineers, Erlangen, Germany) at the University of Cambridge Core Biochemical Assay Laboratory. Samples were also sent for proteomic analysis using a commercially available panel of 92 cardiovascular and inflammatory biomarkers (Olink, Uppsala, Sweden). We selected the cardiovascular III panel which includes markers of endothelial activation and vascular inflammation (https://www.olink.com/products-services/target/cardiovascular-iii-panel/).^22^

### Statistical analysis

We analyzed associations both with average FSS score as a continuous variable and with the presence of fatigue defined as an average FSS score of ⩾ 4.^16,17^ VAF score was inverted to remain consistent with other measures of fatigue (higher scores representing higher fatigue). Methodology by Lopez et al.^21^ was used to create the GDS-F subscale.

Demographics, clinical features, and MRI imaging features were compared between fatigued and non-fatigued groups using logistic regression. Lacune count showed right skew and was normalized with logarithm-10 transformation, while normalized gray matter and WMH volume also showed right skew and were normalized with square root and natural logarithm transformations, respectively.

We assessed PK BP in two ways; first, by measuring the mean binding in the AWM and white matter compartments (NAWM, WMH) and second by determining the hotspots of increased ligand binding. Mean PK BP in the white matter tissues (AWM, NAWM, WMH, EAWM, and ENAWM) was compared between binarized fatigue groups while controlling for age and sex using logistic regression. Correlational analyses tested for association between global BP and any fatigue measure (FSS average score, VAF score, GDS-F score, and binarized fatigue). Hotspots of inflammation were established as voxels of the white matter tissue with a PK ligand uptake of above a 95% percentile value as established in a previous healthy control sample.^22^ The hotspot volume was then divided by the total volume of the corresponding tissue mask to give an overall percentage of voxels showing values above the threshold in each tissue. Hotspots were compared between binarized fatigue groups while controlling for age and sex, with further correlational analyses to test for association between inflammation hotspots in each tissue and fatigue measures. Some Olink biomarkers were skewed and were normalized accordingly (TFF3, Notch3, TR, FAS, CTSD, SCGB3A2, and TR-AP were normalized with logarithmic transformation, CHIT1 and PON3 with square transformation, and MMP-9 and PDGF Subunit A with reflection and then logarithmic transformation).

CRP showed right skew and was normalized using cube root transformation. Five participants had serum CRP scores of less than 0.2, which were converted to 0.2 for analysis. Serum CRP was compared between the binarized fatigue groups using logistic regression while controlling for age and sex. Correlations tested the relationship between CRP and fatigue measures. For all analyses, any significant variables were inputted into both linear (average FSS score) and logistic (binary fatigue groups) regression while controlling for additional covariates.

Olink biomarkers were tested for significant differences between fatigued and non-fatigued groups. A Bonferroni-corrected alpha level of *p* = 0.00054 was implemented to adjust for the large number of multiple comparisons (*n* = 92). To reduce the number of dimensions, principal component analysis (PCA) was carried out; the first principal component identified was then tested for association with fatigue measures using correlation and regression analyses. All analyses were carried out using R version 4.2.3.

## Data availability

Data from this study are available from the corresponding author on reasonable request.

## Results

Demographics of the cohort as a whole, and divided according to the presence or absence of fatigue, are shown in Table 1. Mean (SD) age was 68.74 (11.16) years and 63.9% were male. Mean (SD, range) time from last stroke was 20 months (24.0, 1–93). Average FSS fatigue score (M (SD)) was 4.02 (1.73), average VAF score was −6.63 (2.40), and average GDS-F score was 3.21 (2.68). All fatigue measures were positively and significantly correlated with one another (all *p*s < 0.001).

**Table 1.** Table to show demographic comparison of those with and without fatigue on the FSS.

	Whole cohort (*n* = 36)	No fatigue (*n* = 16)^†^	Fatigue (*n* = 20)^†^	Comparison of *p*-value
Age (M, SD, min–max)	68.74, 11.16, 50.3–87.8	70.38, 11.06, 50.3–87.8	67.3, 11.58, 52.1–87.5	0.43
Sex (% male)	23 (63.89)	10 (62.5)	13 (65)	0.10
Cardiovascular risk factors				
Diabetes mellitus type 1 (% yes)	1 (2.78)	0 (0)	1 (5.0)	0.99
Diabetes mellitus type 2 (% yes)	6 (16.67)	2 (12.50)	4 (20.0)	0.60
History of hypercholesterolemia (% yes)	27 (75.0)	11 (68.75)	16 (80.0)	0.56
History of hypertension (% yes)	31 (86.11)	12 (75.0)	18 (90.0)	0.61
Ischemic heart disease (% yes)	4 (11.11)	2 (12.50)	2 (10.0)	0.93
History of ever smoking (% yes)	21 (58.33)	9 (56.25)	12 (60.0)	0.97
Medications				
Aspirin (% yes)	17 (47.22)	9 (56.25)	8 (40.0)	0.36
Clopidogrel (% yes)	17 (47.22)	7 (43.75)	10 (50.0)	0.70
Dipyridamole (% yes)	1 (2.78)	1 (6.25)	0 (0)	0.99
NOAC (% yes)	0 (0)	0 (0)	0 (0)	NA
Statin (% yes)	32 (88.89)	15 (93.75)	17 (85.0)	0.43
ACE inhibitor (% yes)	14 (38.89)	4 (25.0)	10 (50.0)	0.18
Alpha blocker (% yes)	6 (16.67)	3 (18.75)	3 (15.0)	0.79
Angiotensin II receptor antagonists (% yes)	6 (16.67)	5 (31.25)	1 (5.0)	**0.05**
Beta blocker (% yes)	2 (5.56)	1 (6.25)	1 (5.0)	0.99
Calcium channel blocker (% yes)	21 (58.33)	9 (56.25)	12 (60.0)	0.93
Diuretic (% yes)	5 (13.89)	3 (18.75)	2 (10.0)	0.47
Other antihypertensive (% yes)	1 (2.78)	1 (6.25)	0 (0)	0.99
Clinical features				
Number of prior TIAs (M, SD, min–max)	1.38, 0.92, 0–3	1.33, 0.58, 1–2	1.5, 0.84, 1–3	0.98
Number of prior strokes (M, SD, min–max)	1.44, 0.73, 1–3	1.6, 1.14, 0–3	1, 0, 1–1	0.1
NIHSS score at last stroke (median, IQR, min–max)^†^	3, 2.5, 0–13	3, 2.5, 0–13	3, 2.25, 0–11	0.72
History of migraine (% yes)	11 (30.56)	3 (20.0)	8 (40.0)	0.20
History of depression (% yes)^*^	10 (27.78)	1 (6.67)	9 (45.05)	**0.04**
Total GDS score (median, IQR, min–max)^*^	6, 11.5, 0–28	4.5, 5, 0–21	13, 10.75, 0–28	**0.02**
mRS score (median, IQR, min–max)^*^	1, 1, 0–2	0, 1, 0–1	1, 0, 0–2	**0.02**
Neuroimaging features (M, SD, min–max)				
CMB count^†^	4.32, 14.19, 0–82	2.69, 4.74, 0–16	5.78, 19.13, 0–18	0.63
Lacune count^†^	2.59, 1.99, 0–8	2.25, 1.81, 0–6	2.89, 2.14, 1–8	0.56
Normalized total brain volume (mm^3^)^†^	1,430,380.37, 76,343.98, 1,316,298.62–1,639,121.55	1,439,655.30, 40,389.05, 1,366,114.28–1,507,716.04	1,422,136.00, 98,598.52, 1,316,298.62–1,639,121.55	0.21
Normalized gray matter volume (mm^3^)^†^	698,844.11, 43,494.68, 620,102.44–795,840.62	702,828.64, 37,714.46, 628,312.56–782,804.16	695,302.31, 48,877.67, 620,102.44–795,840.62	0.34
Normalized white matter volume (mm^3^)^†^	731,536.26, 50,901.93, 617,748.11–843,280.93	736,826.66, 30,956.35, 664,077.66–777,841.66	726,833.68, 64,298.43, 617,748.11–843,280.93	0.40
WMH volume (mm^3^)^†^	37,397.46, 25,893.14, 5667.21–103,197.08	31,693.50, 25,763.39, 10,383.72–103,197.07	35,495.95, 26,819.08, 5667.21–94,767.21	0.48

SD: standard deviation; NIHSS: National Institutes of Health Stroke Scale; IQR: Interquartile range; GDS: Geriatric Depression Scale; CMB: Cerebral Microbleed; FSS: Fatigue Severity Scale; mRS: Modified Rankin Scale; WMH: white matter hyperintensities.

*Note*. ^*^p < 0.05, ^**^p < 0.01.

† Indicates missing data (see eTable 1). Excluding age and sex, all *p-*values are adjusted for age and sex.

Notably, 20 subjects (55.6%) had fatigue as defined by an FSS score ⩾ 4. Fatigued and non-fatigued participants did not differ in age, sex, CVRFs, history of stroke, or any neuroimaging feature (Table 1). There were more people in the non-fatigued group taking Angiotensin-II receptor antagonists (*p* = 0.03, Table 1). Fatigued participants had higher history of depression rates (*p* = 0.04), GDS scores (*p* = 0.02), and mRS scores (*p* = 0.02) (Table 1).

### CNS inflammation and fatigue

There were no associations between any fatigue measure and mean PK BP in any white matter tissue. Spearman’s rank correlation coefficients for eroded and non-eroded masks were as follows—average FSS score: EAWM: ρ = 0.07, *p* = 0.71; ENAWM: ρ = −0.28, *p* = 0.14; AWM: ρ = −0.18, *p* = 0.38; NAWM: ρ = −0.26, *p* = 0.190; WMH: ρ = 0.04, *p* = 0.85; VAF score: EAWM: ρ = −0.11, *p* = 0.58; ENAWM: ρ = −0.09, *p* = 0.64, AWM: ρ = −0.03, *p* = 0.88; NAWM: ρ = 0.02, *p* = 0.91; WMH: ρ = −0.06, *p* = 0.76; and GDS-F score: EAWM: ρ = −0.17, *p* = 0.39; ENAWM: ρ = −0.18, *p* = 0.36; AWM: ρ = −0.11, *p* = 0.61; NAWM: ρ = −0.03, *p* = 0.88; WMH: ρ = −0.17, *p* = 0.41.

Similarly, there was no correlation between any fatigue measure and microglial hotspots. Spearman’s rank correlation coefficients for eroded and non-eroded masks, respectively, were EAWM (average FSS: ρ = 0.04, *p* = 0.83; VAF: ρ = −0.29, *p* = 0.12; GDS-F: ρ = −0.20, *p* = 0.30), ENAWM (average FSS: ρ = −0.18, *p* = 0.35; VAF: ρ = −0.30, *p* = 0.11; GDS-F: ρ = −0.17, *p* = 0.38), AWM (average FSS: ρ = −0.03, *p* = 0.86; VAF: ρ = −0.10, *p* = 0.62; GDS-F: ρ = 0.03, *p* = 0.87), NAWM (average FSS: ρ = −0.07, *p* = 0.71; VAF: ρ = −0.08, *p* = 0.69; GDS-F: ρ = 0.05, *p* = 0.82), and WMH (average FSS: ρ = 0.02, *p* = 0.91; VAF: ρ = −0.03, *p* = 0.86; GDS-F: ρ = −0.03, *p* = 0.87). Additional correlational analyses run to test the association of mean PK BP and microglial hotspots with fatigue while controlling for mRS score also showed no association (eTable 2).

There were no significant differences between fatigued and non-fatigued individuals in mean PK BP, or microglial hotspots, in any white matter tissue (Table 2).

**Table 2.** Comparison of mean PK binding and microglial hotspots between those with and without fatigue.

	No fatigue (*n* = 16)^†^	Fatigue (*n* = 20)^†^	*p*
Mean PK binding (M (SD))			
Eroded masks			
Global PK BP in EAWM	−0.06663 (0.0212)	−0.0630 (0.0287)	0.65
Global PK BP in ENAWM	−0.03646 (0.0228)	−0.04289 (0.0262)	0.48
Masks			
Global PK BP in AWM	−0.07181 (0.0112)	−0.07041 (0.0350)	0.75
Global PK BP in NAWM	−0.06244 (0.0122)	−0.06388 (0.0351)	0.4
Global PK BP in WMH	−0.15530 (0.0462)	−0.1474 (0.0500)	0.86
Microglial hotspots (M (SD))			
Eroded masks			
PK BP hotspot volume of EAWM (%)	7.17 (2.43)	7.84 (3.07)	0.48
PK BP hotspot of ENAWM (%)	10.17 (3.67)	10.16 (5.13)	0.94
Masks			
PK BP hotspot volume of AWM (%)	6.74 (2.36)	7.47 (4.71)	0.82
PK BP hotspot of NAWM (%)	7.35 (2.45)	7.98 (5.33)	0.92
PK BP hotspot of WMH (%)	1.71 (1.89)	1.74 (1.75)	0.77

PK: ^11^C-PK11195; SD: standard deviation; BP: binding potential; EAWM: eroded total white matter; ENAWM: eroded normal-appearing white matter; WMH: white matter hyperintensities; AWM: total white matter; NAWM: normal-appearing white matter.

*Note. n* = 30, ^*^p < 0.05, ^**^p < 0.01.

† Indicates missing data (see eTable 1). All *p-*values controlled for age and sex.

### Peripheral inflammation and fatigue

**Figure 1.**
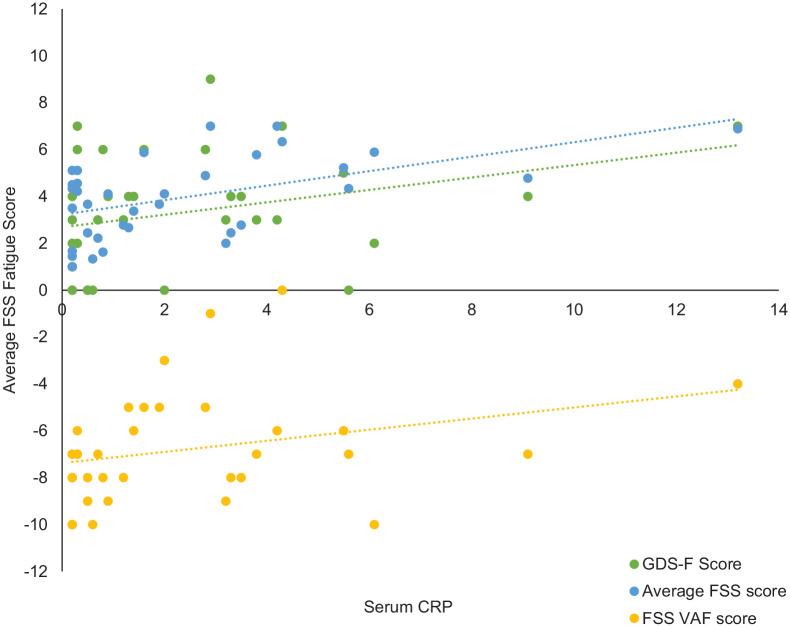
Scatterplot to show the relationship between fatigue measures and serum CRP.

Overall, 34 (94.44%) participants had blood analyses available. There was a significant correlation between serum CRP and average FSS score (ρ = 0.48, *p* = 0.004), VAF score (ρ = 0.37, *p* = 0.03), and GDS-F score (ρ = 0.36, *p* = 0.04) (Figure 1). Average FSS and VAF scores remained associated after controlling for age and sex (ρ = 0.43, *p* = 0.01 and ρ = 0.36, *p* = 0.04, respectively), while GDS-F score was borderline (ρ = 0.34, *p* = 0.055). Based on multiple regression, while controlling for disability (mRS) and history of depression, CRP remained associated with average FSS score (β = 0.49, *p* = 0.03) (Table 3).

**Table 3.** Table to show linear regression testing association of serum CRP with fatigue while controlling for confounders.

Linear regression: average FSS score					
	*df*	Sig	Standardized β	95% CI for β	
				Lower	Upper
(Intercept)	1	0.873	0.428	−4.774	5.628
Age	1	0.920	1.22	−0.049	0.054
Sex	1	0.334	0.003	−0.485	1.463
Serum CRP^*^	1	0.031	0.489	0.174	2.258
History of depression^*^	1	0.015	1.535	0.375	2.695
Modified Rankin Scale score^*^	1	0.021	0.919	0.188	1.650

FSS: fatigue severity scale; CI: confidence interval; CRP: C-reactive protein.

*Note. n* = 35, ^*^p < 0.05, ^**^p < 0.01. Model *R*^2^ = 0.54.

Mean (SD) serum CRP was significantly higher in the fatigued group than the non-fatigued group (3.34 (3.48) vs 1.23 (1.15), *p* = 0.05), but significance was lost when controlling for age and sex (*p* = 0.09).

Sensitivity analyses were carried out to control for potential active infections, by removing all cases of with a CRP level of >10. This resulted in the removal of one case, after which analyses showed all CRP results remained the same as above.

There were no significant differences in any Olink biomarker between the fatigued and non-fatigued groups at the Bonferroni-corrected alpha level of *p* = 0.00054 (see eTable 3 for individual results).

PCA identified several components; the first principal component (PC1) explained 28.61% of variance in the data. PC1 was not correlated with any fatigue measure (average FSS: ρ = −0.07, *p* = 0.71; VAF: ρ = −0.10, *p* = 0.58; GDS-F: ρ = −0.21, *p* = 0.22), but was significantly and negatively correlated with serum CRP scores (ρ = −0.33, *p* = 0.03).

## Discussion

Fatigue is a common symptom in SVD, but the role of inflammation in its pathogenesis is uncertain. In this cohort with moderate-to-severe symptomatic SVD, we found an association between fatigue and peripheral inflammation as evidenced by blood CRP levels, but no association was found with central inflammation as measured using PK PET.

One hypothesis has been that fatigue in SVD, and after stroke more broadly, is associated with increased and persisting CNS inflammation. However, there is little data on this in SVD, largely due to difficulty of measuring CNS inflammation. One marker of CNS inflammation is PK ligand binding on PET, which represents microglial activation.^22^ We found no evidence of any association between ligand binding and fatigue. Results were consistent when looking at different white matter compartments and when using eroded measurements to reduce the risk of partial volume from inadvertent inclusion of abnormal tissue, such as that in white matter lesions or lacunes. We were unable to find any previous studies using PK PET to investigate fatigue in SVD or stroke.

In contrast to the lack of association with central inflammation, circulating CRP levels were significantly associated with fatigue severity, even when controlling for age, sex, depression, and mRS as additional confounders. However, there was no association between binarized fatigue and CRP. In addition, biomarkers from the Olink cardiovascular III panel were not associated with the presence or severity of fatigue. Our results suggest that systemic inflammation may be a risk factor for fatigue, but further data from larger cohorts are required.

Previous studies have shown mixed results on the relationship between CRP and fatigue. Some have suggested an association,^8,30,31^ although two found the relationship disappeared after controlling for confounders.^9,32^ With the exception of one large study (*n* = 212),^30^ most have been small to moderate in size (all < 100)^8,9,31–33^ and larger studies with careful controlling for confounding covariates are required.

Previous studies of other circulating inflammatory biomarkers in stroke have also given conflicting results. One study in 45 post-stroke participants found higher IL-1β predicted fatigue at 6 months and lower IL-1ra and IL-9 predicted fatigue at 12 months,^8^ this study assessed biomarkers < 72 hours after stroke, whereas we assessed a cohort who were at least 3-month post-acute stroke, which may account for the differences seen in our results. However, another study (*n* = 327) also failed to replicate this and, after controlling for confounders, only showed associations with stem cell factor, which further lost association after correction for multiple comparisons.^34^ Differences in between results may also be attributable to the different methods used; while the aforementioned studies assessed cytokines, we evaluated a proteomics panel.

In contrast to the lack of association with central inflammation, and possible associations with systemic inflammation, we found associations between fatigue and both history of depression and current GDS score, and current disability. This is consistent with the previous findings.^4,35–37^ There were no associations with age, sex, or any CVRF.

Our study has several strengths. Most importantly, to our knowledge, this is the first study to investigate whether CNS inflammation is associated with fatigue in SVD using PK PET. We used a standardized PET-imaging protocol and collected fatigue data using multiple standardized and validated fatigue measures. Measurements of fatigue severity and PK were performed blinded to each other.

However, it also has limitations. Although the sample size was reasonable for a PET study, it was relatively small, reducing power to detect associations, thus, it may be fruitful to replicate these findings in a larger cohort to confirm the results in a higher-powered study. However, we found no trend toward any associations between PK ligand binding and fatigue, and a post hoc analysis suggested we would need a sample size of 392 in each group to detect any difference, suggesting that it is unlikely we were underpowered to detect a difference in PK binding between the two groups. We also used several measures of fatigue; however, these may not fully capture the multifaceted nature of fatigue.^5,38^ In addition, the MINERVA study did not collect data on physical activity, which can influence both fatigue and inflammation, and therefore, we were unable to control for this. Future research should further investigate the role of inflammation, particularly CRP, with fatigue in SVD in larger cohorts while using fatigue measures that consider the multidimensional aspects of fatigue and controlling for other confounders, such as physical activity.

We used PK to measure CNS inflammation, as this is a validated marker and also allows spatial inflammation on the pattern of microglial activation. Cerebrospinal fluid was not available, but it would be interesting to obtain this to assess classical markers of brain inflammation, such as TNF-α, IL-1β, NO, or COX 2. While we measured a wide variety of serum inflammatory markers, these were limited to CRP and those on the cardiovascular Olink panel. These did not include some established markers of systemic inflammation, such as RNA-binding protein, TNFα, and HSP70.

Finally, although PK PET is widely used as a marker of microglial activation, it may be confounded by off-target and non-specific tissue binding;^39,40^ we controlled for these where possible by correcting for vascular endothelial binding in the analysis. It is also true that not all cells involved in the inflammatory process, such as cytokines and leukocytes, are reflected by the PET tracer and this may reduce sensitivity of the imaging to neuroinflammation.^41^ Lastly, a recent transcriptomic study suggested that TSPO relates to microglial concentration rather than phenotype.^42^ Accordingly, it may be the case that PK signal represents a combination of pro- and anti-inflammatory cells that are not deleterious when accounted together.

In conclusion, we found no evidence for a role of CNS inflammation in the pathogenesis of fatigue. We found support for a possible role of systemic inflammation, but further studies are required to understand this relationship and determine whether it could be therapeutically modified to reduce fatigue severity.

## Supplemental Material

sj-docx-1-wso-10.1177_17474930241245613 – Supplemental material for Are central and systemic inflammation associated with fatigue in cerebral small vessel disease? Supplemental material, sj-docx-1-wso-10.1177_17474930241245613 for Are central and systemic inflammation associated with fatigue in cerebral small vessel disease? by Amy A Jolly, Robin B Brown, Daniel J Tozer, Young T Hong, Tim D Fryer, Franklin I Aigbirhio, John T O’Brien and Hugh S Markus in International Journal of Stroke
