# Supplementary material for: Are central and systemic inflammation associated with fatigue in cerebral small vessel disease?
Source: Int J Stroke. 2024 Apr 12;19(6):705–13. doi: 10.1177/17474930241245613 (PMC11292988; doi:10.1177/17474930241245613)
Supplement: sj-docx-1-wso-10.1177_17474930241245613 – Supplemental material for Are central and systemic inflammation associated with fatigue in cerebral small vessel disease? [file sj-docx-1-wso-10.1177_17474930241245613.docx]

**Supplementary Material**

eTable 1. Table to show missing data for the current analyses.

eTable 2. Table to show correlation between PET measures and fatigue measures whilst controlling for mRS score.

eTable 3. Olink blood biomarker comparison between fatigued and non-fatigued groups.

***eTable 1.* Table to show missing data for the current analyses.**

|  | **Available** | **Missing** | **Total** |
| --- | --- | --- | --- |
| GDS | 35 | 1 | 36 |
| FSS | 36 | 0 | 36 |
| FSS VAF | 35 | 1 | 36 |
| MRI | 34 | 2 | 36 |
| Brain Volume | 34 | 2 | 36 |
| CMB | 34 | 2 | 36 |
| Lacunes | 34 | 2 | 36 |
| WMH | 26 | 10 | 36 |
| PET | 30 | 6 | 36 |
| EWM | 30 | 6 | 36 |
| ENAWM | 30 | 6 | 36 |
| Lesion | 30 | 6 | 36 |
| AWM | 30 | 6 | 36 |
| NAWM | 30 | 6 | 36 |
| CRP | 35 | 1 | 36 |
| Olink Biomarkers | 35 | 1 | 36 |
| *Note.* Total recruited to MINERVA trial *n*=44, total with FSS fatigue testing *n*=36. | | | |

***eTable 2.* Table to show correlation between PET measures and fatigue measures whilst controlling for mRS score.**

|  | FSS Average Score | VAF score | GDS-F score |
| --- | --- | --- | --- |
| *Mean PK BP* | | | |
| EAWM | ρ=-0.08, *p=*0.70 | ρ=-0.15, *p=*0.45 | ρ=-0.21, *p=*0.30 |
| ENAWM | ρ=-0.31, *p=*0.11 | ρ=-0.10, *p=*0.62 | ρ=-0.22, *p=*0.29 |
| AWM | ρ=-0.12, *p=*0.56 | ρ=0.03, *p=*0.87 | ρ=-0.008, *p=*0.97 |
| NAWM | ρ=-0.20, *p=*0.33 | ρ=0.08, *p=*0.70 | ρ=0.01, *p=*0.95 |
| WMH | ρ=-0.04, *p=*0.86 | ρ=-0.09, *p=*0.65 | ρ=-0.11, *p=*0.58 |
| *Microglial Hotspots* | | | |
| EAWM | ρ=-0.03, *p=*0.87 | ρ=-0.36, *p=*0.07 | ρ=-0.18, *p=*0.37 |
| ENAWM | ρ=-0.19, *p=*0.34 | ρ=-0.31, *p=*0.12 | ρ=-0.19, *p=*0.34 |
| AWM | ρ=-0.08, *p=*0.68 | ρ=-0.06, *p=*0.76 | ρ=-0.08, *p=*0.69 |
| NAWM | ρ=-0.05, *p=*0.82 | ρ=-0.03, *p=*0.88 | ρ=-0.10, *p=*0.63 |
| WMH | ρ=0.10, *p=*0.62 | ρ=-0.002, *p=*0.99 | ρ=-0.03, *p=*0.90 |
| *Note. Spearman Rank* coefficients and corresponding *p*-values given. *= *p*<0.05, **= *p*=0.00054. | | | |

***eTable 3.* Olink blood biomarker comparison between fatigued and non-fatigued groups.**

| Biomarker (M, SD) | No Fatigue    (*n=*16) † | Fatigue    (*n=*20) † | Comparison | Age & sex controlled |
| --- | --- | --- | --- | --- |
| ALCAM | 7.36 (0.22) | 7.39 (0.26) | *p=*0.70 | *p=*0.62 |
| AP_N | 5.96 (0.28) | 6.03 (0.26) | *p=*0.50 | *p=*0.77 |
| AXL | 8.52 (0.33) | 8.50 (0.27) | *p=*0.81 | *p=*0.73 |
| AZU1 | 6.21 (1.18) | 6.45 (1.58) | *p=*0.62 | *p=*0.74 |
| BLM hydrolase | 3.51 (0.28) | 3.47 (0.42) | *p=*0.75 | *p=*0.52 |
| CASP-3 | 6.13 (0.78) | 6.42 (1.15) | *p=*0.39 | *p=*0.55 |
| CCL15 | 7.83 (0.37) | 7.62 (0.48) | *p=*0.18 | *p=*0.23 |
| CCL16 | 7.12 (0.48) | 7.29 (0.68) | *p=*0.41 | *p=*0.40 |
| CCL24 | 5.23 (0.78) | 5.71 (0.89) | *p=*0.13 | *p=*0.18 |
| CD163 | 7.76 (0.36) | 7.99 (0.51) | *p=*0.16 | *p=*0.12 |
| CD93 | 10.94 (0.20) | 10.84 (0.27) | *p=*0.25 | *p=*0.31 |
| CDH5 | 3.83 (0.40) | 3.71 (0.33) | *p=*0.32 | *p=*0.34 |
| CHI3L1 | 6.49 (0.55) | 7.05 (1.17) | *p=*0.09 | *p=*0.07 |
| CHIT1 | 4.51 (2.43) | 4.43 (2.21) | *p=*0.56 | *p=*0.62 |
| CNTN1 | 3.15 (0.40) | 3.16 (0.32) | *p=*0.97 | *p=*0.99 |
| COL1A1 | 2.16 (0.45) | 2.06 (0.32) | *p=*0.44 | *p=*0.49 |
| CPA1 | 6.06 (0.63) | 6.07 (0.47) | *p=*0.97 | *p=*0.96 |
| CPB1 | 6.25 (0.58) | 6.24 (0.51) | *p=*0.96 | *p=*0.95 |
| CSTB | 3.58 (0.25) | 3.79 (0.67) | *p=*0.24 | *p=*0.24 |
| CTSD | 3.58 (0.25) | 3.79 (0.67) | *p=*0.24 | *p=*0.24 |
| CTSZ | 4.45 (0.42) | 4.45 (0.53) | *p=*0.98 | *p=*0.80 |
| CXCL16 | 5.86 (0.23) | 5.91 (0.34) | *p=*0.61 | *p=*0.58 |
| DLK-1 | 6.70 (0.63) | 6.86 (0.62) | *p=*0.48 | *p=*0.32 |
| EGFR | 3.13 (0.21) | 3.07 (0.22) | *p=*0.51 | *p=*0.15 |
| Ep CAM | 5.01 (1.00) | 5.58 (0.92) | *p=*0.09 | *p=*0.11 |
| EPHB4 | 4.63 (0.26) | 4.52 (0.38) | *p=*0.31 | *p=*0.38 |
| FABP4 | 3.85 (0.80) | 3.86 (0.96) | *p=*0.97 | *p=*0.91 |
| FAS | 6.56 (0.23) | 6.66 (0.57) | *p=*0.57 | *p=*0.47 |
| Gal-3 | 5.00 (0.30) | 5.01 (0.41) | *p=*0.94 | *p=*0.87 |
| Gal-4 | 4.71 (0.53) | 4.86 (0.69) | *p=*0.47 | *p=*0.54 |
| GDF-15 | 4.94 (0.67) | 5.01 (0.72) | *p=*0.77 | *p=*0.45 |
| GP6 | 3.30 (0.80) | 3.66 (0.82) | *p=*0.19 | *p=*0.22 |
| GRN | 5.40 (0.23) | 5.55 (0.37) | *p=*0.18 | *p=*0.22 |
| ICAM-2 | 5.36 (0.34) | 5.36 (0.42) | *p=*0.98 | *p=*0.91 |
| IGFBP-1 | 5.57 (0.63) | 5.10 (1.49) | *p=*0.25 | *p=*0.21 |
| IGFBP-2 | 8.54 (0.71) | 8.16 (0.63) | *p=*0.10 | *p=*0.17 |
| IGFBP-7 | 8.30 (0.25) | 8.10 (0.44) | *p=*0.11 | *p=*0.15 |
| IL-17RA | 3.56 (0.50) | 3.91 (0.63) | *p=*0.56 | *p=*0.12 |
| IL-18BP | 6.14 (0.31) | 6.18 (0.37) | *p=*0.75 | *p=*0.62 |
| IL-1RT1 | 5.31 (0.26) | 5.27 (0.29) | *p=*0.64 | *p=*0.69 |
| IL-1RT2 | 5.54 (0.24) | 5.56 (0.44) | *p=*0.87 | *p=*0.73 |
| IL2-RA | 4.39 (0.52) | 4.51 (0.56) | *p=*0.52 | *p=*0.35 |
| IL-6RA | 12.44 (0.24) | 12.32 (0.28) | *p=*0.18 | *p=*0.11 |
| ITGB2 | 5.42 (0.34) | 5.50 (0.37) | *p=*0.51 | *p=*0.53 |
| JAM-A | 5.39 (0.31) | 5.52 (0.49) | *p=*0.36 | *p=*0.49 |
| KLK6 | 3.78 (0.26) | 3.73 (0.38) | *p=*0.66 | *p=*0.75 |
| LDL Receptor | 4.48 (0.56) | 4.70 (0.71) | *p=*0.31 | *p=*0.32 |
| LTBR | 3.15 (0.33) | 3.07 (0.45) | *p=*0.55 | *p=*0.58 |
| **MB*** | 7.88 (0.48) | 7.51 (0.59) | *p=*0.05* | *p=*0.05* |
| MCP-1 | 5.55 (0.43) | 5.56 (0.41) | *p=*0.99 | *p=*0.97 |
| MEPE | 4.54 (0.36) | 4.42 (0.43) | *p=*0.36 | *p=*0.30 |
| MMP-2 | 2.05 (0.25) | 1.96 (0.21) | *p=*0.27 | *p=*0.38 |
| **MMP-3*** | 6.61 (0.54) | 6.08 (0.68) | *p=*0.02* | *p=*0.01* |
| MMP-9 | 5.99 (0.69) | 6.11 (1.10) | *p=*0.41 | *p=*0.47 |
| MPO | 4.25 (0.80) | 4.56 (0.85) | *p=*0.29 | *p=*0.35 |
| Notch 3 | 5.05 (0.47) | 5.03 (0.30) | *p=*0.89 | *p=*0.80 |
| NT proBNP | 2.86 (1.23) | 2.75 (0.91) | *p=*0.75 | *p=*0.81 |
| OPG | 3.63 (0.25) | 3.55 (0.45) | *p=*0.52 | *p=*0.73 |
| OPN | 7.47 (0.49) | 7.34 (0.45) | *p=*0.42 | *p=*0.58 |
| PAI | 7.67 (0.37) | 7.87 (0.58) | *p=*0.26 | *p=*0.34 |
| PCSK9 | 2.49 (0.21) | 2.62 (0.41) | *p=*0.29 | *p=*0.28 |
| PDGF Subunit A | 5.04 (0.66) | 5.31 (0.60) | *p=*0.13 | *p=*0.18 |
| PECAM-1 | 4.30 (0.28) | 4.33 (0.36) | *p=*0.80 | *p=*0.92 |
| PGLYRP1 | 8.41 (0.51) | 8.70 (0.77) | *p=*0.21 | *p=*0.21 |
| PI3 | 2.91 (0.71) | 3.09 (0.66) | *p=*0.43 | *p=*0.31 |
| PLC | 7.90 (0.26) | 7.80 (0.32) | *p=*0.31 | *p=*0.46 |
| PON3 | 6.24 (0.66) | 5.90 (0.84) | *p=*0.21 | *p=*0.19 |
| PRTN3 | 4.56 (0.91) | 5.01 (1.21) | *p=*0.26 | *p=*0.26 |
| PSP-D | 2.45 (0.60) | 2.31 (0.60) | *p=*0.48 | *p=*0.58 |
| RARRES2 | 11.88 (0.23) | 11.96 (0.33) | *p=*0.40 | *p=*0.49 |
| RETN | 6.24 (0.32) | 6.36 (0.64) | *p=*0.49 | *p=*0.45 |
| **SCGB3A2*** | 2.83 (1.07) | 2.11 (0.54) | *p=*0.01* | *p=*0.03* |
| SELE | 11.28 (0.53) | 11.55 (0.77) | *p=*0.24 | *p=*0.37 |
| SELP | 10.42 (0.62) | 10.63 (0.70) | *p=*0.36 | *p=*0.37 |
| SHPS-1 | 3.51 (0.38) | 3.40 (0.40) | *p=*0.39 | *p=*0.34 |
| SPON1 | 0.47 (0.28) | 0.41 (0.33) | *p=*0.55 | *p=*0.57 |
| ST2 | 4.24 (0.54) | 3.97 (0.49) | *p=*0.14 | *p=*0.09 |
| TFF3 | 5.06 (0.41) | 4.96 (0.35) | *p=*0.43 | *p=*0.52 |
| TFPI | 8.96 (0.38) | 8.94 (0.38) | *p=*0.88 | *p=*0.84 |
| TIMP4 | 3.63 (0.36) | 3.44 (0.71) | *p=*0.33 | *p=*0.49 |
| **TLT-2*** | 5.03 (0.31) | 5.37 (0.32) | *p=*0.003* | *p=*0.01* |
| TNF-R1 | 6.95 (0.31) | 6.96 (0.43) | *p=*0.95 | *p=*0.84 |
| TNF-R2 | 5.36 (0.33) | 5.47 (0.44) | *p=*0.42 | *p=*0.37 |
| TNFRSF10C | 6.18 (0.45) | 6.21 (0.50) | *p=*0.84 | *p=*0.78 |
| TNFRSF14 | 5.33 (0.28) | 5.45 (0.48) | *p=*0.36 | *p=*0.35 |
| TNFSF13B | 6.89 (0.19) | 7.04 (0.37) | *p=*0.14 | *p=*0.13 |
| t-PA | 5.00 (0.54) | 5.35 (0.71) | *p=*0.11 | *p=*0.13 |
| TR | 4.23 (0.45) | 4.28 (0.89) | *p=*0.86 | *p=*0.56 |
| **TR-AP*** | 4.84 (0.30) | 5.16 (0.47) | *p=*0.02* | *p=*0.04* |
| uPA | 5.12 (0.26) | 5.04 (0.30) | *p=*0.40 | *p=*0.30 |
| U-PAR | 6.07 (0.39) | 6.13 (0.58) | *p=*0.72 | *p=*0.71 |
| **vWF*** | 6.19 (0.48) | 6.52 (0.47) | *p=*0.04* | *p=*0.03* |
| *Note*. 𝛼 level of 0.00054 to correct for multiple comparisons. *= *p*<0.05, **= *p=*0.00054. | | | | |
